# Supplementary material for: HIVprotI: an integrated web based platform for prediction and design of HIV proteins inhibitors
Source: J Cheminform. 2018 Mar 9;10:12. doi: 10.1186/s13321-018-0266-y (PMC5845081; doi:10.1186/s13321-018-0266-y)
Supplement: Supplementary file 2 — Additional file 2. Source code of HIVProtI web server. [file 13321_2018_266_MOESM2_ESM.zip › HIVprotI_Source-code/hivprotI/weka-3-6-6/doc/weka/core/pmml/DerivedFieldMetaInfo.html]

DerivedFieldMetaInfo


---


|  |  |  |
| --- | --- | --- |
| **Overview** \| **Package** \| **Class** \| **Tree** \| **Deprecated** \| **Index** \| **Help** \| **Weka's home** | |  |
| **PREV CLASS**   **NEXT CLASS** | **FRAMES**    **NO FRAMES**     **All Classes** |  |
| SUMMARY: NESTED \| FIELD \| CONSTR \| METHOD | DETAIL: FIELD \| CONSTR \| METHOD |  |


---


## weka.core.pmml Class DerivedFieldMetaInfo

```
java.lang.Object
  weka.core.pmml.FieldMetaInfo
      weka.core.pmml.DerivedFieldMetaInfo
```

**All Implemented Interfaces:**: java.io.Serializable

---

``` public class DerivedFieldMetaInfo extends FieldMetaInfo implements java.io.Serializable ```

**See Also:**: Serialized Form

---

**Nested Class Summary**

**Nested classes/interfaces inherited from class weka.core.pmml.FieldMetaInfo**

`FieldMetaInfo.Interval, FieldMetaInfo.Optype, FieldMetaInfo.Value`


**Constructor Summary**

`DerivedFieldMetaInfo(org.w3c.dom.Element derivedField,
java.util.ArrayList<Attribute> fieldDefs,
weka.core.pmml.TransformationDictionary transDict)`


| **Method Summary** | |
| --- | --- |
| `double` | `getDerivedValue(double[] incoming)`             Get the derived field value for the given incoming vector of values. |
| `Attribute` | `getFieldAsAttribute()`             Get this derived field as an Attribute. |
| `void` | `setFieldDefs(java.util.ArrayList<Attribute> fieldDefs)`             Upadate the field definitions for this derived field |
| `java.lang.String` | `toString()` |

**Methods inherited from class weka.core.pmml.FieldMetaInfo**

`getFieldName, getOptype`

**Methods inherited from class java.lang.Object**

`equals, getClass, hashCode, notify, notifyAll, wait, wait, wait`

**Constructor Detail**

### DerivedFieldMetaInfo

```
public DerivedFieldMetaInfo(org.w3c.dom.Element derivedField,
                            java.util.ArrayList<Attribute> fieldDefs,
                            weka.core.pmml.TransformationDictionary transDict)
                     throws java.lang.Exception
```

**Throws:**: `java.lang.Exception`


**Method Detail**

### setFieldDefs

```
public void setFieldDefs(java.util.ArrayList<Attribute> fieldDefs)
                  throws java.lang.Exception
```

:   Upadate the field definitions for this derived field

    :   **Parameters:**: `fieldDefs` - **Throws:**: `java.lang.Exception`

---


### getFieldAsAttribute

```
public Attribute getFieldAsAttribute()
```

:   Get this derived field as an Attribute.

    :   **Specified by:**: `getFieldAsAttribute` in class `FieldMetaInfo`
    :   **Returns:**: an Attribute for this derived field.

---


### getDerivedValue

```
public double getDerivedValue(double[] incoming)
                       throws java.lang.Exception
```

:   Get the derived field value for the given incoming vector of
    values. Incoming values are assumed to be in the same order
    as the attributes supplied in the field definitions ArrayList
    used to construct this DerivedField.
    If the optype of this derived field is continuous, then a real
    number is returned. Otherwise, the number returned is the index
    of the categorical/ordinal value corresponding to result of computing
    the derived field value.

    :   **Parameters:**: `incoming` - the incoming parameter values **Returns:**: the result of computing the derived value **Throws:**: `java.lang.Exception` - if there is a problem computing the value

---


### toString

```
public java.lang.String toString()
```

:   **Overrides:**: `toString` in class `java.lang.Object`


---


|  |  |  |
| --- | --- | --- |
| **Overview** \| **Package** \| **Class** \| **Tree** \| **Deprecated** \| **Index** \| **Help** \| **Weka's home** | |  |
| **PREV CLASS**   **NEXT CLASS** | **FRAMES**    **NO FRAMES**     **All Classes** |  |
| SUMMARY: NESTED \| FIELD \| CONSTR \| METHOD | DETAIL: FIELD \| CONSTR \| METHOD |  |


---
